# Does precrastination explain why some observers are suboptimal in a visual search task?

**DOI:** 10.1098/rsos.191816

**Published:** 2024-04-24

**Authors:** Alasdair D. F. Clarke, Anna Nowakowska, Kyle Sauerberger, David A. Rosenbaum, Thomas R. Zentall, Amelia R. Hunt

**Affiliations:** ^1^ Department of Psychology, University of Essex, Colchester, UK; ^2^ School of Psychology and Vision Sciences, University of Leicester, Leicester, UK; ^3^ Department of Psychology, University of California, Riverside, CA, USA; ^4^ Department of Psychology, University of Kentucky, Lexington, KY, USA; ^5^ School of Psychology, University of Aberdeen, Aberdeen, UK

**Keywords:** optimal behaviour, visual search, eye movements

## Abstract

How do we decide where to search for a target? Optimal search relies on first considering the relative informational value of different locations and then executing eye movements to the best options. However, many participants consistently move their eyes to locations that can be easily ascertained to neither contain the target nor provide new information about the target’s location. Here, we asked whether this suboptimal search behaviour represents a specific example of a general tendency towards precrastination: starting sub-goals of a task before they are needed, and in so doing, spending longer time on doing the task than is necessary. To test this hypothesis, we asked 200 participants to do two tasks: retrieve two heavy buckets (one close and one far) and search for a line segment. Precrastination is defined as consistently picking up the closer bucket first, versus the more efficient strategy of picking up the farther bucket first. Search efficiency is the proportion of fixations directed to more cluttered regions of the search array. Based on the pilot data, we predicted an association of precrastination with inefficient search strategies. Personality inventories were also administered to identify stable characteristics associated with these strategies. In the final dataset, there was no clear association between search strategy and precrastination, nor did these correlate strongly with any of the personality measures collected. This article received in-principle acceptance (IPA) at *Royal Society Open Science* on 29 January 2020. The accepted Stage 1 version of the manuscript, not including results and discussion, may be found at https://osf.io/p2sjx. This preregistration was performed prior to data collection and analysis.

## 1. Introduction

Human behaviour in visual search tasks is characterized by large individual differences. While both optimal [1,2] and stochastic [3] strategies have been proposed, experiments show that different observers use different strategies [4,5], with some approximating the optimal search strategy, others behaving randomly and others still, making use of a ‘counter-optimal’ strategy. The challenge for a complete understanding of visual search is to identify the source(s) of these individual differences.

**Figure 1.**
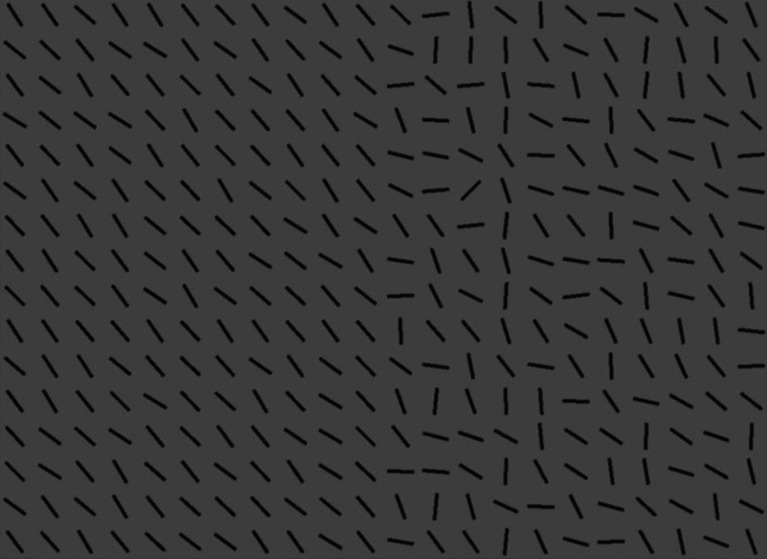
An example of the split-half line segment stimuli. The target is the unique line segment oriented 45° to the right. Participants who direct their saccades to the heterogeneous half of the display are faster in correctly responding to target’s absence/presence than those who fixate the homogeneous half of the display.

In the *split-half* visual search paradigm [4], the observer is presented with a stimulus similar to that shown in figure 1. The observer’s task is to decide whether the target (a line segment oriented 45° to the right) is present or not, as quickly and accurately as possible. One side of the display contains distractors of a similar orientation, and the other contains more variable orientations. In this situation, the optimal strategy is to use central vision to search the side of the stimulus containing the heterogeneous orientations: if the target were present on the more homogeneous side of the display, the observer would be able to use peripheral vision to see it immediately. Moving the eyes towards the homogeneous side provides no new information and slows down search considerably (indeed, in Nowakowska *et al*. [6], each fixation on the homogeneous side was estimated to slow response times by 360 ms). While approximately a third of observers implement an optimal strategy, a similar number pursued the opposite strategy and searched the easy (homogeneous) side of the display first, despite this leading to slower response times.

Departures from optimality have also been found in other visual search tasks, such as Irons and Leber’s [5,7] task, in which two unique targets are present and participants need to find only one of them. Predictable changes to the distractors make one target easier to find than the other. A subset of participants showed optimal performance, in that they searched for the easier target, given the set of distractors on that trial. However, the majority of participants did not switch between targets in a way that was related to changes in the distractors. Curiously, while both Irons and Leber’s and the split-half line segment paradigms appear to have good test–retest correlations ( r≈0.75 ), there is, at best, only a weak correlation between the two [8]. This suggests that it is not the case that some people are ‘good at looking for things’ while others are not. Instead, something about the structure of the particular task leads some people to perform well and others to perform badly.

Previous attempts have similarly failed to link behaviour during visual search tasks with broader personality and individual difference measures. Irons and Leber found no correlation with search strategy for working memory, impulsiveness, novelty-seeking, intolerance of uncertainty or need for cognition^1^ [5,7]. Similarly, Jóhannesson *et al*. found no evidence of an effect of working memory or inhibitory control on foraging behaviour [9]. Given that these search tasks appear to be uncorrelated, a general trait is unlikely to explain variability in all search tasks.

In the present study, we investigated the hypothesis that participants who are suboptimal in our split-half line segment search task have a tendency towards *precrastination*, a phenomenon where participants prioritize the completion of a sub-goal, even if it results in extra effort overall [10]. In the original study, participants were asked to walk down a corridor, pick up one of two heavy buckets as they passed and carry it to the finish line. The two buckets were placed at different distances between the start and finish lines, and the optimal (in terms of minimizing effort) strategy is to pick up the bucket that is furthest from the starting position and closer to the finish line. Despite this simple solution, most participants favoured picking up the first bucket they came to, even though that resulted in them having to carry it further. This tendency was named *precrastination* and has since been replicated several times in both humans [11] and pigeons [12]. In an even more dramatic version of the task, Fournier *et al*. [11] asked participants to go get two buckets—one close and one further away—and bring them back to set them on a table behind them. The majority (36 out of 49 participants in the key condition) chose to pick up the close bucket first, carry it out to the further bucket, and then bring both back, even though this order is clearly more effortful than picking up the far bucket and then grabbing the close one on the way back to the table.

As a first step to assessing our hypothesis, we conducted a pilot study to ensure that we would replicate the variability between participants in a shorter version of the search task and that we could clearly classify people into two groups based on whether they pick up the close bucket first (i.e. ‘precrastinators’) or the farther bucket (optimal). The pilot data were also intended to be used to refine our approach to data processing, visualization and analysis. Both the previous findings (search variability [4] and precrastination [11]) were clearly replicated in the pilot experiment. Surprisingly, the data suggested a relatively robust relationship between precrastination and visual search strategy but in the opposite direction to the one which we originally expected, with precrastinators having a tendency to search more efficiently rather than less. We, therefore, revised our aim to be more data-driven than the original hypothesis, aiming to estimate the variability in search performance accounted for by precrastination. The pilot data also suggested that the relationship between precrastination and visual search may be mediated by differences in speed–accuracy trade-offs between participants, so we planned to explore this aspect of visual search as well.

If we confirm the existence of a relationship between these two tasks, a natural question is whether this can be accounted for by personality traits of the individuals. A personality trait is an enduring pattern of thoughts, feelings and behaviours that is stable across time and contexts [13]. Previous research suggests that those who precrastinate are motivated, responsible and energetic [14]. Specifically, Sauerberger [15] found a correlation of *r* = 0.22 between precrastinaion and conscientiousness, which we attempted to replicate. Speculatively, these traits may also cause participants to search in a more thorough but less efficient manner, aligning with the interpretation that precrastinating, although costly, may not be irrational to the degree that participants prefer maximizing thoroughness over maximizing efficiency. Personality traits in this study were examined to help further uncover the individual difference factors associated with precrastination and different visual search strategies.

Our experiment tested the hypothesis that precrastination might represent a rational approach to doing tasks, that is, a reasonable tendency to get the difficult part of the task out of the way first. If precrastination had been positively related to efficient search, this would be evidence in favour of a ‘rational’ interpretation of precrastination, as opposed to the alternative interpretation that it reflects an irrational impulse to grab the closest task-related object (in which case, a negative correlation with search efficiency would have been more likely). In addition, the experiment had the potential to assess whether the already-established link between precrastination and conscientiousness could generalize and be a potential explanation for the wide range of different search strategies previously observed. This would have important implications for theories of visual search, which tend to make assumptions about how ‘people’ (as a group) direct their eyes during search; if personality traits can be directly predictive of eye movement strategies, this would challenge the field to more carefully refine its assumptions. In addition to the clear theoretical contribution our results could have had, they could also have illustrated a case where efficient/optimal tendencies in one circumstance can translate to inefficient choices in others.

## 2. Pilot results

Data from 30 participants were analysed as part of our pilot study. These were classed as either precrastinating (*n* = 13) or not (*n* = 17) based on their behaviour in the bucket task, as described in §2.1. We now investigate the extent to which this classification of participants is correlated with the variance observed in the split-half visual search task.

### 2.1. Accuracy and reaction time

**Figure 2.**
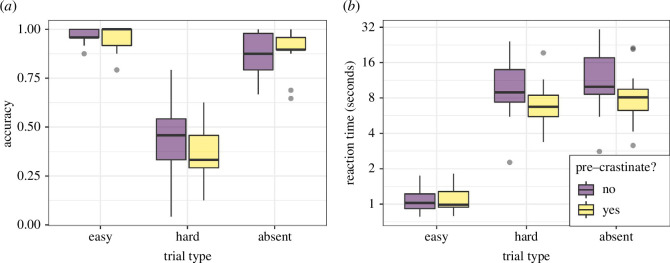
The distribution of (*a*) accuracy and (*b*) reaction time measures in the split-half visual search task. Note that the *y*-axis for (*b*) has been log_2_-scaled. Dots represent points classed as outliers, defined as being below or above 1.5× the interquartile range from the first or third quartile.

Each participant’s median reaction time and accuracy were computed for the split-half visual search task, presented in figure 2. It appears that the group of participants who precrastinated in the bucket task found fewer hard targets in the visual search task (median accuracy of 0.33) than the participants who did not precrastinate (median 0.46). We can also see that the precrastinators had shorter median response times: *easy*: 0.99 versus 1.03 s; *hard*: 6.71 versus 8.91 s; and *absent*: 8.08 versus 9.95 s. These results suggest varying speed–accuracy trade-offs between our two groups, with participants labelled as precrastinators being more likely to give up before finding the hard targets.

### 2.2. Search strategy

**Figure 3.**
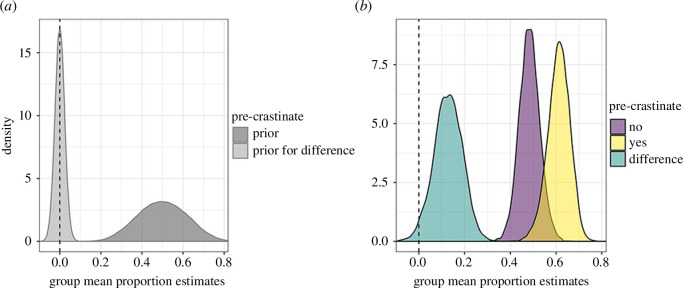
(*a*) Prior and (*b*) posterior distributions for the mean of the two groups of participants and the difference between them. Our priors for the two groups are identical, hence the overlapping distributions.

To investigate the relationship between a participant’s tendency to precrastinate and how they approach the visual search task, we fit beta distributions^2^ to the strategy metric for each group. The strategy metric is defined as the proportion of fixations two through five directed to the heterogeneous half of the display. We use a Bayesian approach with a conservative prior for the difference in group means. We assume that the mean of each group is 0.50, with a 95% highest posterior density interval (HPDI) = [0.27, 0.73]. Our prior for the difference between groups is conservative, β∼N(0,0.50) , which gives a distribution with a 95% HPDI = [−0.047, 0.046]. These distributions are illustrated in figure 3*a*.

After conditioning the model on the data, we obtain posterior probability distributions shown in figure 3*b*. Our best estimate for the difference in group means is 0.125, with a 95% HPDI of [−0.003, 0.242]. An alternative summary would be that *p*(*δ *> 0 | *X*) = 0.973.^3^

### 2.3. Discussion and hypotheses for registration

Based on the results of our pilot analysis, it appears that there is a relationship between precrastination and visual search strategy; participants who did not precrastinate had a mean value of 0.5 for their strategy metric, while those who did precrastinate searched more optimally, with a mean strategy metric of around 0.625. Interestingly, this goes against our original intuition in which we viewed precrastination as suboptimal behaviour that may explain the suboptimal behaviour in our search paradigm: people who needlessly pick up the closer bucket would be the same people who needlessly search the homogeneous half of the display. The pilot data support the opposite conclusion that suboptimal precrastination leads to more optimal behaviour in our visual search task. Upon reflection, participants who show a desire to start their task sooner may be starting the search task sooner as well, by targeting the heterogeneous regions where effortful search is required. If confirmed, this relationship would reinforce the interpretation that precrastination does not indicate suboptimal decision strategies but instead indicates a conscientious approach to task performance.

As such, we investigated the following hypotheses:

1. There is a difference (in either direction) in search behaviour between those participants who precrastinated and those who did not. This will be determined by looking at p(δ>0|X) . A value of <0.05 will be taken as evidence that precrastination leads to less optimal search strategies, while a value of >0.95 will be taken as evidence for the opposite.
2. The magnitude of the difference is in line with the value of 0.125 (95% HPDI of [−0.003, 0.242]) obtained in our pilot experiment. This will be determined by comparing the new HPDI with the pilot HPDI. A new estimate for the size of the difference will then be computed by pooling all data in a mixed-effect model (with random effects for data collected in Aberdeen, Essex and Pilot).
3. There is a positive correlation (*r* = 0.22) between precrastination and conscientiousness.

## 3. Methods

We planned to recruit 200 participants for this experiment. This large sample size is due to the addition of personality measures: larger samples provide greater power to detect the typical effect sizes in those fields, reduce Type I error and increase the chances of replicable findings [16]. Participants were recruited via participant panels and opportunity sampling at the universities of Aberdeen and Essex. The protocol was approved by the ethics committees at both universities. Participants completed the bucket-retrieval task both before and after the visual search task, so no counterbalancing was needed. Additional participants were recruited to replace those excluded from data analysis (details below).

A one-tailed correlation power analysis (using the pwr package [17]) with α=0.05,β=0.8 and *n* = 200 gives *r* = 0.175.

### 3.1. Precrastination

#### 3.1.1. Apparatus and stimuli

**Figure 4.**
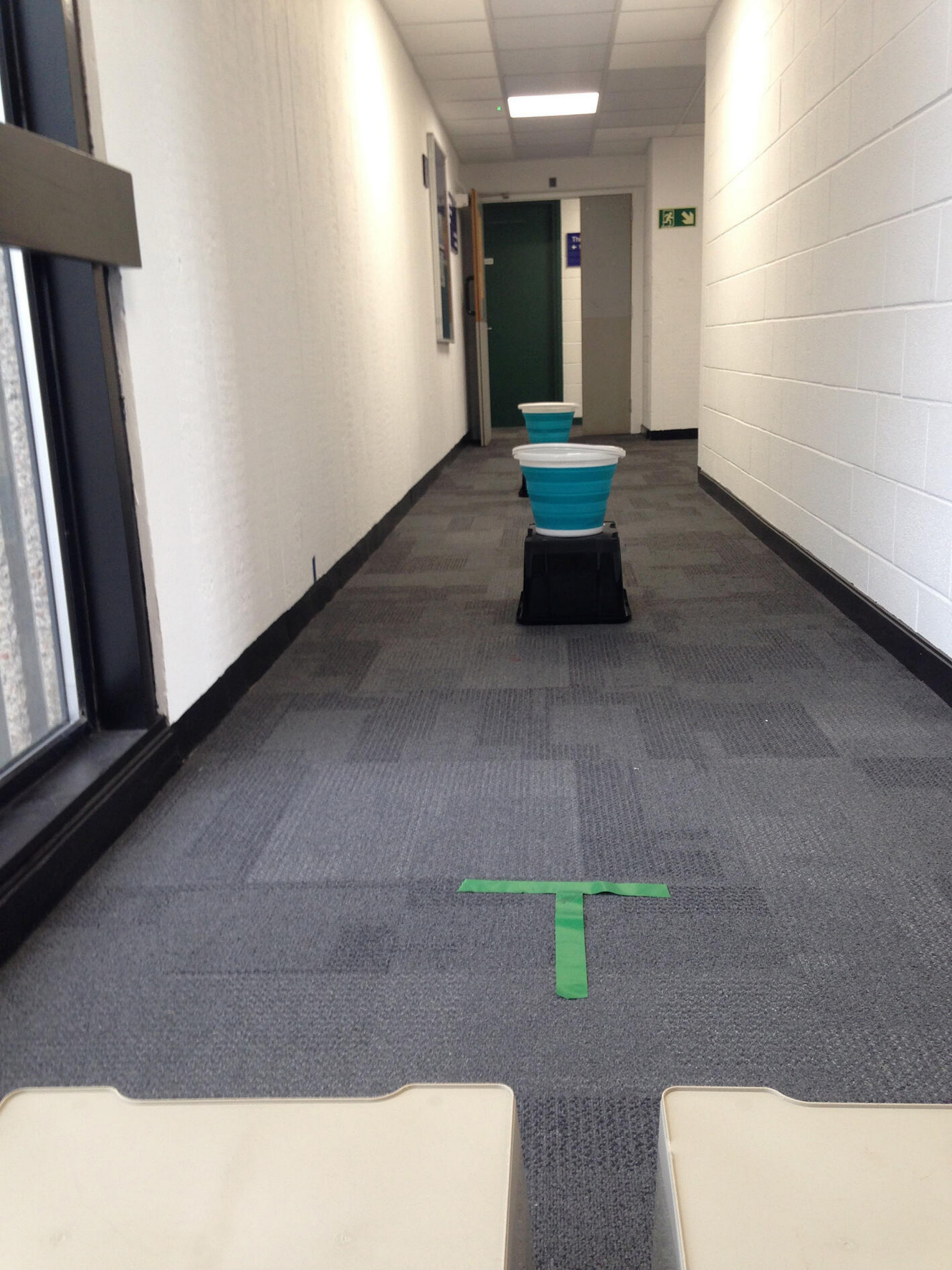
Set-up for the precrastination part of the experiment.

Figure 4 is a photograph of the bucket task set-up. Two 10 l buckets were filled with sand until the total weight of each bucket was 3 kg. The experiment took place in a quiet corridor. Buckets were placed at 6 and 16 ft away from the start position, which was marked as a T with green tape. Buckets were placed on crates at about hand height. Just behind the start position was a pair of crates on which to set the buckets down to end the trial.

#### 3.1.2. Procedure

The task replicates that used in Fournier *et al*. [11], in which participants were instructed to pick up two buckets and place them on the crates behind the start position. We closely followed the methods and instructions as described in their appendix. Each participant completed three trials before the visual search task and three trials after. While the experimenter was setting up the buckets for the next trial, the participant was asked to walk over to a nearby window and look outside until asked to return. The full protocol and instructions given to participants are available at https://osf.io/5aq4c/.

#### 3.1.3. Data processing

Participants were classified as ‘precrastinators’ if they picked up the closer bucket first and ‘optimal’ if they picked up the far bucket first. Based on the pilot data, and that of Fournier *et al*. [11], we expected the results from the bucket task to be largely binary, with participants picking up either the close or the far bucket on every trial. Our plan was the following: if the data are similarly binary in this experiment, we will continue to treat precrastination as a two-level categorical factor. Participants who pick up the closest bucket on at least five of the six trials will be classed as precrastinators, while those who pick up the closest bucket on no more than two out of six trials will be non-precrastinators. Participants with intermediate behaviour will be excluded.

There was also a chance that, unlike Fournier *et al*. [11], our sample of participants would give rise to a larger range of intermediate behaviours. If more than 25% of our participants would have been excluded under the coding scheme outlined above, then we would have treated our precrastination measure as a three-level factor (if a clear classification is apparent). In this case, the same Bayesian beta regression model would have been fitted to the data.

### 3.2. Split-half visual search

#### 3.2.1. Apparatus and stimuli

Most of the data were collected at the University of Aberdeen. Stimuli were presented on a 17-inch CRT monitor with a resolution of 1024 × 768. Around a third of participants were tested at the University of Essex using a 21-inch LCD panel with a resolution of 1920 × 1080. The display distance was set so that the visual angle of the stimuli was approximately the same between the two sites. Stimulus generation, presentation and data collection were controlled by Matlab and the psychophysics toolbox [18,19] that run on a Mac Pro computer. The position of the right eye was recorded using a desktop-mounted EyeLink 1000 eye tracker (SR Research, Canada), sampling eye position at 1000 Hz.

**Figure 5.**
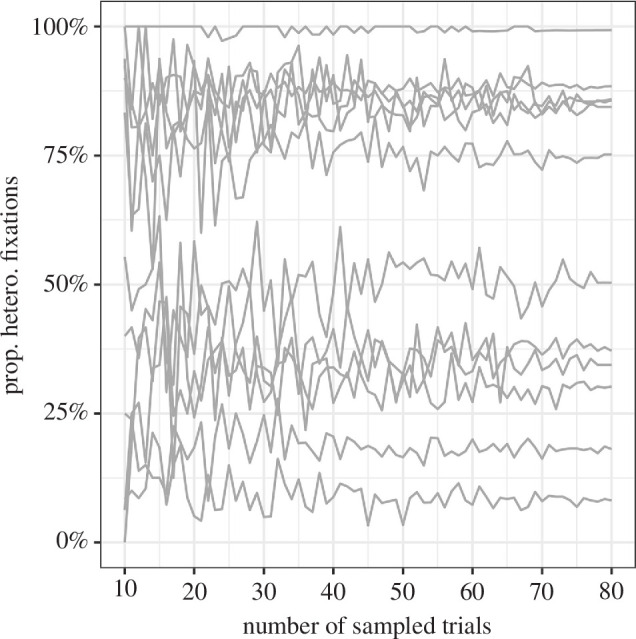
The effect of reducing the number of target-absent trials on measurement accuracy. Each line represents one of the participants from Nowakowska *et al*. [4], and at the far right, we can see the measurement of their strategy based on all 80 target-absent trials. If we only use a random sample of 48 trials per participant, then we still obtain approximately the same proportion.

We used the same arrays of line segments as in Nowakowska *et al*. [4] (figure 1). These arrays consist of 22 columns and 16 rows on a uniform grey background. The target line is always tilted 45° to the right, while the mean distractor angle is perpendicular at 45° to the left. Search difficulty was manipulated by sampling from either a narrow 30° range of distractor line orientations (homogeneous) or a wide 106° range (heterogeneous). Half of each search array consists of homogeneous line segments, while the other half is heterogeneous. The side with heterogeneous segments was randomly determined for each trial. We used 96 search trials in total, half of which contained a target. This number of trials was selected based on simulating the results of shorter experiments using the data from Nowakowska *et al*. [4] (figure 5). The target could be located in any of the possible locations apart from the middle four vertical columns.

#### 3.2.2. Procedure

Before starting the visual search experiment, participants undergo a nine-point calibration sequence. Following this, participants were instructed to report, as quickly as possible, whether a line tilted 45° to the right was present or not, using the up (present) or down (absent) arrow key. Each search trial begins with a black fixation point (letter x) subtending 1.5 cm × 2.5 cm (1.9° × 3.1°), presented at the centre of the computer screen. On the press of a space bar, the stimulus is displayed until the participant makes a response (or times out after 60 s). Auditory feedback in the form of a beep and a 2-s red screen immediately follows incorrect key presses. A set of eight practice trials precedes the experimental trials.

#### 3.2.3. Data processing

Data from participants who failed to achieve at least 75% accuracy for *easy* and *absent* conditions were excluded as it suggests that they did not understand the task. Fixations during trials were classified as falling on the homogeneous or heterogeneous half of the display, with fixations falling within the central four columns of line segments being excluded. Following Nowakowska *et al*. [4], we summarized each participant’s strategy as the proportion of fixations two through five falling on the heterogeneous side of the display during target-absent trials. Trials with fewer than five fixations were excluded, and we only analysed data from participants who have at least 10 trials.

### 3.3. Personality questionnaires

#### 3.3.1. Materials

We administered three personality questionnaires. The first questionnaire is the second iteration of the Big Five Inventory (BFI-2) [20]. The BFI-2 measures five broad personality traits: extraversion (e.g. ‘is outgoing, sociable’), agreeableness (e.g. ‘has a forgiving nature’), conscientiousness (e.g. ‘is efficient, gets things done’), negative emotionality (e.g. ‘can be tense’) and open-mindedness (e.g. ‘is fascinated by art, music or literature’). Based on previous research, we suspected that precrastination will have the strongest relationship with conscientiousness. Those who are high in conscientiousness are organized, productive and responsible and may have an urge to pick up a bucket as soon as possible.

The second personality questionnaire is the Barratt Impulsiveness Scale (BIS-11) [21]. The BIS-11 measures overall impulsiveness and three subtypes: attentional impulsiveness (‘I don’t “pay attention”’), motor impulsiveness (‘I act on the spur of the moment’) and non-planning impulsiveness (‘I say things without thinking’). Based on previous research, we suspected that neither impulsiveness nor its subscales will be related to precrastination.

The third questionnaire is the procrastination scale [22]. The procrastination scale was created specifically to use in student populations and measures a broad range of procrastination behaviours. Sample items include ‘I often find myself performing tasks that I had intended to do days before’, ‘I generally delay before starting on work I have to do’ and ‘I usually have to rush to complete a task on time’. Procrastination is delaying a task or decision with the knowledge that doing so will impair progress towards a goal; it is irrational. Therefore, pre- and procrastination should be unrelated.

#### 3.3.2. Procedure

Personality questionnaires were administered to participants at the end of the experiment to prevent them from influencing the behavioural measures. Questionnaires were hosted on Qualtrics and participants accessed the questionnaires using a laboratory computer.

Participants were allowed to skip questions they did not wish to answer. For any one questionnaire, a response rate of less than 75% of questions or giving the same response for more than 75% of all questions, invalidated the data from that questionnaire. Participants would be replaced if they answered less than 75% of the questions on the conscientiousness scale (the others are exploratory).

### 3.4. Planned analysis

The aim of this experiment is to determine if there is a relationship between precrastination and the suboptimal behaviour that we observe in the split-half visual search task. Following Nowakowska *et al*. and Clarke *et al*. [4,8], each participant’s strategy was represented by the proportion of fixations two through five directed to the difficult half of the search area. We used a Bayesian approach to estimate the extent to which performance in the precrastination bucket task is predictive of visual search strategy. Our approach to Bayesian modelling followed the advice given by McElreath [23], with models fitted using R [24] and Stan [25].

As our independent variable is a proportion, we fitted a model using beta regression [26] with a logit link for μ (mean) and a log link for ϕ (precision). We used conservative priors: β∼N(0,0.50) and γ∼N(0,1) (where β and γ are the coefficients for μ and γ , respectively). This means that we are assuming that the mean of each group is 0.50, with a 95% HPDI = [0.27, 0.73].^4^ Our prior for the difference between groups is β∼N(0,0.50) . We have included pilot data (above) to demonstrate our planned analysis. We have previously used this analysis approach with this paradigm to explore the effect of motivation on visual search strategy [27].

We also measured the relationship between the personality measures and tendency to precrastinate, using similar methods to those outlined above. If our measures of precrastination turned out to be mainly binary, which they did, then we planned to use Bayesian logistic regression to measure the relationship. Otherwise, we would have reported an *R* ^2^ statistic.

### 3.5. Outcome-neutral conditions

The ability to test the stated hypotheses depends on replicating both the precrastination behaviour and the variability in visual search efficiency observed in previous studies. Our pilot data achieve this replication. Given that we planned to repeat the same conditions with a larger sample, it is unlikely that we would not be able to test our hypotheses. Nonetheless, in our pilot results, the proportion of participants who were categorized as precrastinators (13/30) was smaller than in the most similar previous experiment (Fournier *et al*. [11], 36/49). Some variation in the proportion can be expected, but a very high (>90%) or very low (<10%) prevalence of precrastination would have suggested there is something in the way we set up or instructed the bucket task that provoked an unrepresentative behaviour in our participants and would not be able to use these data to test our hypotheses. It was also critical that we find inter-participant variability in search strategy. Our pilot data replicate this variation as well, and it was difficult to imagine why variability would be restricted in our larger sample. Nonetheless, if the variability in our sample had been less than 0.1 (= half the standard deviation of the pilot data (0.2)), we would not have proceeded to use these data to test our hypotheses.

### 3.6. Exploratory analysis

The main (pre-registered) hypothesis motivating this experiment is that participants who precrastinate will also be suboptimal (in terms of making more early eye movements to the homogeneous half of the display) in the split-half visual search task. After carrying out our pilot experiment, it looked like there may also be a difference in speed–accuracy trade-offs. If this (or any other differences in eye movements) had appeared to be an interesting effect after we collected our full dataset, we would have carried out suitable exploratory analysis. We also investigated the possibility of further links between the personality measures, precrastination and visual search.

## 4. Results

We collected data from a grand total of 305 participants. Fourteen of these participants had to be removed due to missing eye-tracking data. Out of the 291 remaining, 90 failed to meet our inclusion criteria for the visual search task.^5^ This leaves us with 201 participants with visual search data. The precrastination task was completed by 303 participants (1 participant was excused from this part of the experiment because of a broken leg and another for an unspecified reason). The personality questionnaires were completed by 304 participants (20 participants had blank or otherwise unusable data).

### 4.1. Visual search

**Figure 6.**
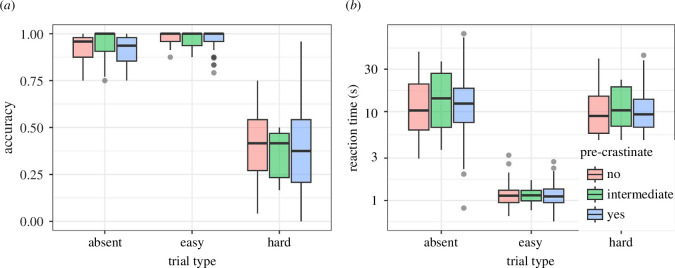
The distribution of (*a*) accuracy and (*b*) reaction time measures in the split-half visual search task. Note that the *y*-axis for (*b*) has been log_2_-scaled. Dots represent points classed as outliers, defined as being below or above 1.5x the interquartile range away from the first or third quartile.

**Figure 7.**
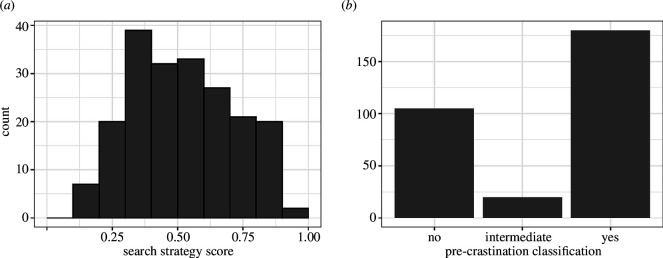
The distribution of (*a*) search strategy efficiency scores and (*b*) precrastination behaviours. We can see that there is a lot of variation in both measures.

Each participant’s median reaction time and accuracy were computed for the split-half visual search task, as shown in figure 6. The results are broadly in line with those that we have seen in previous uses of this paradigm. More importantly, we see a large variation in search strategy, as shown in figure 7*a*, replicating previous results. The standard deviation of our efficiency score is 0.192.

### 4.2. Precrastination

The range of precrastination behaviours is shown in figure 7*b*. The majority of participants clearly either precrastinated (180, 59.0%) or not (105, 34.4%) with only a small minority (20, 6.5%) following an intermediate strategy. As such, we will treat precrastination as a two-level factor and exclude participants exhibiting intermediate behaviours.

### 4.3. Planned analysis

**Figure 8.**
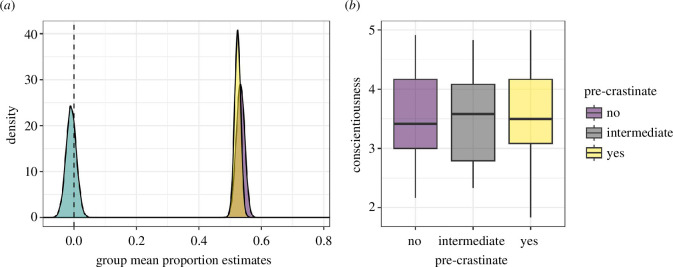
(*a*) Posterior distributions for the mean of the two groups of participants and the difference between them. (*b*) There is no evidence for a relationship between precrastination and conscientiousness.

The first step of our planned analysis is to test the hypothesis that there is a difference in visual search strategy between those participants who precrastinated and those who did not. As registered, we fitted a Bayesian beta regression model, the results of which are displayed in figure 8. There is little-to-no evidence for a difference between the two groups (*p*(*δ *> 0|*X*) = 0.50). This lack of differences makes hypothesis 2 (that the effect size will be in line with the pilot data) redundant, but we report that the 95% HPDI = [−0.0465, 0.0467] for the sake of completeness.

Our final registered hypothesis concerns the relationship between precrastination and conscientiousness. We find no evidence for this relationship in our data. This holds whether we continue to treat precrastination as a binary variable (95% HPDI = [−0.19, 0.17] for the difference between groups) or as a continuous variable and measure the number of trials out of six in which participants precrastinated (95% HPDI = [−0.03, 0.03] for the effect of number of trials on conscientiousness).

#### 4.3.1. Discussion

We met all outcome-neutral conditions and acquired a sample sufficient in size and quality to test the three hypotheses. As with the pilot data, the larger sample demonstrated that precrastination behaviour is common, with more than half the participants picking up the close bucket first. We also replicated the wide range of variation between individuals in visual search efficiency. We found no evidence for a consistent relationship between visual search efficiency and precrastination, however, and no relationship between either measure and conscientiousness. Therefore, no evidence in favour of our registered hypotheses resulted from this experiment.

### 4.4. Exploratory analysis

We now investigate whether our data suggest any new hypotheses that could be of interest to other researchers.

#### 4.4.1. Personality and precrastination

**Figure 9.**
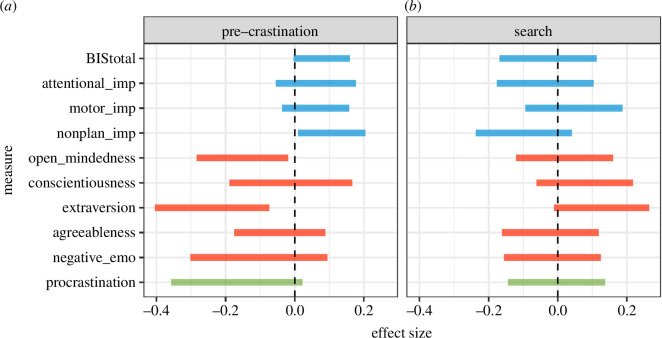
(*a*) Relationship between the precrasination and each of the personality measures. The effect size measures the 95% confidence interval for the mean difference between participants who precrastinated and those who did not. (*b*) Correlations between our search strategy metric and the personality measures. The effect size measures the 95% confidence interval in Pearson’s *r*.

The left column of figure 9 shows the relationship between 10 personality measures and precrastination in a sample of 304 participants. The width of the bar is the range of the 95% CI around the difference between precrastinators and non-precastinators on each measure. We find some tentative evidence to suggest that participants who precrastinate may score slightly higher in impulsivity and lower in extraversion and open-mindedness. We did not make any specific predictions about these personality measures, and so we refrain from interpreting these, but report them here for the benefit of future research, with the caution that many of the effect size estimates cover a range that includes or is close to 0, even with this large sample.

#### 4.4.2. Personality and visual search strategy

The right column of figure 9 shows the relationship between 10 personality measures and visual search strategies, expressed as a correlation between efficiency score (the proportion of fixations on the heterogeneous side) and score on each scale/subscale. We find no evidence of any clear relationships between the personality measures and visual search strategy in this sample of 201 participants.

#### 4.4.3. Other measures

In addition to the registered measures, for efficiency, we collected a range of other demographic characteristics and free-report measures from our participants. These are outside the scope of the questions we have addressed in this paper. A full exploration of these additional variables and their relationship to visual search and precrastination will be presented elsewhere. In the interest of open science, however, and to avoid possible confusion by separating or duplicating data, the full set of data analysed in this pre-registration is presented along with these additional variables here: https://osf.io/5aq4c/ .

## 5. General discussion

Although the pilot data suggested participants who precrastinate tended to use their peripheral vision to search more efficiently, the registered study with a much larger sample did not support that conclusion. While this is a null finding, it is nonetheless meaningful because we had a clear prediction based on data and theory and designed a robust experiment to test it, which underwent rigorous peer review. Both the pilot and the final experiment replicated the previously established patterns in finding (1) a good split of people who consistently precrastinated and people who consistently did not and (2) a broad range of individual differences in visual search efficiency. If a stable relationship between these factors exists and is large enough to build any theories around, this experiment would have detected it. The fact that it did not detect it is useful in ruling out explanations for either behaviour (precrastination or visual search inefficiency) that is grounded in a shared personality characteristic like conscientiousness or impulsivity.

The relationship between conscientiousness and precrastination observed previously [15] was not observed here. This could be because the original effect was a statistical accident or the effect is not present in our sample. One point to note is that our sample was skewed towards the higher end of the conscientiousness scale, likely because unconscientious people are less inclined to participate in psychology experiments. It is possible that the sample in Sauerberger [15] was more representative of the full range of conscientiousness in the population and was, therefore, more sensitive to the relationship with precrastination. On the other hand, one might expect that our more conscientious sample would have higher precrastination rates than previously observed, and this was not the case. Another interesting and counterintuitive observation from Sauerberger [15] was that procrastination was unrelated to precrastination, and the current results show a small negative correlation, as one would expect if starting tasks too soon is predictive of not starting tasks too late. Given the inconsistency of this finding between the present results and Sauerberger’s [15], however, both of which are samples of over 300 participants, the relationship between these two measures is not strong.

Our results provide a relatively definitive answer to a set of related questions: can we explain variation in individual strategies in precrastination and visual search efficiency using these well-established personality questionnaires? Although the simple answer is ‘no’ (or at least, not much), the hypothesis that personality traits can explain or predict behaviour in these tasks cannot be completely put to rest by our results, because it is possible that the relationships are nonlinear, or there are heterogeneous influences that can drive two people to exhibit the same behaviour but for different reasons. Nonetheless, had we found the predicted relationships in the larger sample, this would have been an important finding, that would have indicated that we can predict how people behave in these tasks based on personality traits (and vice versa). It was, therefore, an important possibility to test, even though the results did not bear out the prediction. Moreover, through this project, we have produced a large and very rich dataset with many other measures included, in addition to those reported here. We invite others to use it in hypothesis-generating exploratory analyses of their choosing. We request that anyone using the data in this repository reference this paper.

## Data Availability

All materials, data and analysis scripts are available at the Open Science Framework [28].

## Footnotes

There was a small statistically significant correlation between the need for cognition and the switch rate.

A distribution that is bounded by (0,1) and hence suitable for modelling proportions.

Comparing the frequentist approach to our Bayesian model, we can see that a *t*-test gives both a less precise, and more positive, estimate of the difference: *t* = 2.66, *df* = 2.75, *p* = 0.013, 95% CI = [0.04, 0.32].

For comparison, the mean strategy score for the participants in Nowakowska *et al*. [4] with *n* = 14 is 0.58, with a range of [0.10, 0.99]. Similarly, Clarke *et al*. [8] (*n* = 57) found a mean of 0.60 with a range of [0.06, 0.98].

Further details available in supplementary materials.
